# 

**Published:** 1907-02

**Authors:** 


### The American Journal of Dental Science

The Official Organ of the Florida, Georgia and South Carolina State Dental Societies

**Third Series**

VOL. XXXVIII FEBRUARY, 1907 NO. 2

EDITORS

WM. GIRD BEECROFT, D. D. S., Madison. Wis. B. H. TEAGUE, D. D. S., Aiken, S. C.

Published on the 10th day of each month.
Subscription price $1.00 per year.
ENTERED AS SECOND-CLASS MATTER, MADISON, WIS.

Address all communications, both business and editorial, to
**The Dental Publishing Company, Madison, Wisconsin**

### NOTICES

Georgia State Dental Society meets at Atlanta May 7, 8, 9.

Nebraska State Dental Society meets at Lincoln May 21, 22, 23.

Kentucky State Dental Association meets at Louisville May 20, 21, 22.

RESOLUTIONS Adopted by the American Society of Orthodontists, New York, Dec. 29, 1907.

*Resolved,* That in the opinion of the members of the American Society of Orthodontists, the practice of paying a commission, honorarium, or any sort of fee, in consideration for the reference of a patient is both unwarrantable and unprofessional; and be it

*Resolved,* That the payment of any such commission, honorarium, or fee, by any member of this Society, shall be sufficient cause for the expulsion of said member, by vote of the Society after conviction; and further be it

*Resolved,* That in case of co-operation in the care of a patient between a general practitioner and Orthodontist, there shall be no division of fees, but each man shall render a separate bill for his personal services.

FREDERICK S. McKAY, *Secretary.*

---

**Dr. Wm. H. Povall of Mt. Morris, N. Y. is giving demonstrations of the Whiteside Bridge Crowns at Buffalo this week. The Whiteside Dental Mfg. Co., of Youngstown, O. are the makers of this as well as of the Whiteside Removable Pin Crown.**

